# Long-Term Effects of Haematopoietic Stem Cell Transplantation after Pediatric Cancer: A Qualitative Analysis of Life Experiences and Adaptation Strategies

**DOI:** 10.3389/fpsyg.2017.00704

**Published:** 2017-05-10

**Authors:** Magali Lahaye, Isabelle Aujoulat, Christiane Vermylen, Bénédicte Brichard

**Affiliations:** ^1^Department of Pediatric Hematology and Oncology, Cliniques Universitaires Saint-Luc, Université catholique de LouvainBrussels, Belgium; ^2^Institute of Health and Society, Université catholique de LouvainBrussels, Belgium; ^3^Institute of Experimental and Clinical Research, Université catholique de LouvainBrussels, Belgium; ^4^Department of Pediatrics, Cliniques Universitaires Saint-LucBrussels, Belgium

**Keywords:** haematopoietic stem cell transplantation, pediatric cancer, long-term effects, coping strategies, survivors

## Abstract

Haematopoietic stem cell transplantation (HSCT) improves the survival rate of children and adolescents with malignant and non-malignant conditions; however, the physical, psychological and social burden of such a procedure is considerable both during and after treatment. The present qualitative study investigated the long-term effects of HSCT after pediatric cancer. Thirty adolescent and young adult (AYA) survivors (*M*_age_ = 23.61 years, *SD* = 5.21) participated in individual interviews and were invited to speak about their life experiences following their treatment and strategies they use to deal with their past medical experiences and the long-term sequelae. Our results showed the presence of ongoing physical and psychosocial consequences of their past illness and its treatments with wide ranging psychosocial impacts, such as affected self-image, social withdrawal, sense of lack of choice, and need for specific attention. Different strategies were reported to overcome these consequences, such as talking about illness, giving a sense to their past medical experiences, and developing meaningful social relationships. Clinical and research implications are also discussed.

## Introduction

An increasing number of children and adolescents with cancer are surviving their illnesses, thanks to considerable improvements in the treatments of certain childhood cancers in recent decades (Gatta et al., 2014). Childhood cancers are typically managed with a combination of treatment modalities (surgery, radiation, chemotherapy, etc.,) depending on factors such as the type and stage of the cancer (Siegel et al., 2012). Among these a notable increase in the use of haematopoietic stem cell transplantation (HSCT) has been observed (Cairo and Heslop, 2008; Clarke et al., 2008), particularly when traditional treatments have failed; after a relapse and less frequently as first-line therapy (Armenian et al., 2011).

Haematopoietic stem cell transplantation undoubtedly improves the survival rate of children and adolescents with some types of cancer (Copelan, 2006), however, the physical, psychological and social burden of such a procedure is considerable both during and after treatment. Physical side effects such as nausea, vomiting, alopecia, skin reactions and aplasia can be expected throughout the various stages of treatment (the conditioning regimen, HSCT, graft-versus-host-disease [GvHD]) and may have an impact on the quality of life (QoL) of affected children and their family (Zecca et al., 2002). Moreover, HSCT requires a long hospitalization during which children may experience isolation (both emotional and social) as well as limitations to age appropriate activities and school attendance. This naturally affects children’s QoL, as well as family functioning and parental well-being (Phipps et al., 2005; Clarke et al., 2008).

Beyond side effects during treatment, AYA survivors of HSCT are also at risk of a number of heterogeneous long-term complications such as chronic conditions, cardiac, pulmonary, endocrine, neurological, and neuropsychological problems (Steffens et al., 2008; Baker et al., 2010a,b; Benmiloud et al., 2010; Jadoul et al., 2011; Beauloye et al., 2013). Although they may not always be life-threatening, these complications can impact the QoL of survivors.

In their review, Clarke et al. (2008) showed that in general QoL of young survivors treated with HSCT is similar or better than norms 6 months to 8 years after transplant and depends on different factors such as QoL before the HSCT; individual resources and family functioning. However, Parsons et al. (2012) underlined that only a few studies have investigated the long-term effects of HSCT on QoL (more than 1 or 2 years after transplant). Of these, Löf et al. (2009) showed that survivors 5 years after transplant reported poorer physical functioning, general health, sexual function and partner relationships than norms. Sanders et al. (2010) also demonstrated that despite certain physical, psychosocial and cognitive complications adult survivors treated with HSCT (follow-up at 16 years) could maintain a satisfactory QoL.

Evidently, there is significant variability in the way that AYAs live with their past illness and its complications. Coping strategies have already been explored during treatment for pediatric cancer (e.g., Hildenbrand et al., 2014). However, to our knowledge, they have never been investigated in adult survivors treated with HSCT. The aim of our study therefore was to identify and evaluate the following:

(1) What are the challenges and difficulties that AYA survivors of HSCT still experience due to their past illness and its complications?
(2) What strategies or resources do AYA survivors of HSCT exploit to help them in dealing with the current implications of their past medical experiences?

Better understanding how AYA survivors live with their past illness, its treatment and consequences has value in improving long-term psychosocial interventions and eventually suggesting adapted psychosocial follow-up for such patients.

Moreover, the present study aims to overcome some methodological limitations highlighted in previous reviews on long-term consequences in pediatric oncology (e.g., Eiser et al., 2000; Patenaude and Kupst, 2005; Parsons et al., 2012). Firstly, most studies assessed physical, psychological, and social impact of cancer in children through generic measures and not through cancer-specific outcome measures. Secondly, empirically validated measures often investigate general coping rather than coping with particular physical complications after specific conditions, such as HSCT in pediatric cancer (Kupst and Bingen, 2006; Hildenbrand et al., 2014). Our qualitative design enables the authors to comprehend outcomes without *a priori* conceptual categories of adjustment or maladjustment whilst taking into account individual and contextual information, which is better elaborated through the use of questionnaires. Thirdly, although authors have previously mentioned that they assessed the long-term consequences of cancer and its treatments, many studies were limited to a short follow-up period and very few were devoted to the very long-term impact of cancer and its treatments. The present study offers a long-term perspective. Finally, although many studies have focused on the complications of HSCT, there is a lack of literature regarding coping strategies to deal with these difficulties. One of our aims was to qualify the resources and adaptation competences that AYA survivors develop in order to integrate their past medical experiences into their daily life.

## Materials and Methods

We conducted a descriptive and exploratory qualitative study based on individual in-depth interviews.

### Participants

The participants were recruited on a voluntary basis. Inclusion criteria were as follows: treatment with HSCT for a childhood cancer, being aged 15 years or older and having been cured for more than 3 years. Thirty interviews constituted our final material for qualitative analyses. Socio-demographic and medical information about participants are presented in **Table 1**.

**Table 1:** Characteristics of the 30 patients grouped according to their initial pathology.

	All patients (*N* = 30)	Haemetalogical disease (*N* = 19)	Solid tumor (*N* = 11)
Sex ratio *M/F* (% of males)	18/12 (60%)	11/8 (58%)	7/4 (64%)
**Age *M* ±*SD***			
Age at diagnosis	7.99 ± 4.58	9.97 ± 3.92	4.57 ± 3.58
At the moment of the interview	23.61 ± 5.21	23.70 ± 5.25	23.43 ± 5.38
Adults	26 (87%)	17 (89%)	9 (82%)
**Marital status**			
Single	19 (63%)	12 (63%)	7 (64%)
In couple	11 (37%)	7 (37%)	4 (36%)
**Profession**			
Student	13 (43%)	8 (42%)	5 (45%)
Employee	10 (33%)	7 (37%)	3 (27%)
Unemployed	7 (23%)	4 (21%)	3 (27%)
**Initial diagnosis**			
Acute lymphoblastic leukemia	7 (23%)	7 (37%)	
Acute myeloblastic leukemia	6 (20%)	6 (32%)	
Chronic myeloblastic leukemia	2 (7%)	2 (10%)	
Non-Hodgkin’s lymphoma	3 (10%)	3 (16%)	
Hodgkin’s disease	1 (3%)	1 (5%)	
Neuroblastoma	7 (23%)		7 (64%)
Ewing’s sarcoma	1 (3%)		1 (9%)
Rhabdomyosarcoma	3 (10%)		3 (27%)
**Type of HSCT**			
Autologous	18	7	11
Allogeneic	11	11	0
Both	1	1	0

Three hundred and fifty-seven children less than 18 years of age received a HSCT in the Department of Pediatric Hematology and Oncology of the Cliniques Universitaires Saint-Luc (Brussels, Belgium), between February 1974 and August 2008. At the time of our study, 125 of these were aged 15 years or older; had been cured for more than 3 years, and were still living in Belgium. They were invited to participate in a multidisciplinary research on the endocrine and metabolic consequences of a pediatric HSCT (Steffens et al., 2008; Benmiloud et al., 2010; Jadoul et al., 2011; Beauloye et al., 2013).

As part of this multidisciplinary research, 48 participants agreed to a research interview conducted by a psychologist. Sixteen of these had a non-malignant condition and therefore were excluded from our study. The remaining 32 received a HSCT for a cancer and as such comprised our final sample. Two participants had to be excluded due to incompletion of the first interview, and because during the second interview the guardian of the minor was the primary respondent.

Ethical approval was obtained from the Ethics Committee of the Cliniques Universitaires Saint-Luc (Brussels, Belgium).

### Procedure

Participants first received a letter explaining the general scope of the study. If they accepted to participate in the study, they were invited to contact the Department of Pediatric Hematology and Oncology to schedule a session of medical assessments, including a psychological consultation. At this appointment, the scope of the research was explained again and the desire to participate was confirmed in writing. In the cases of minors, consent forms were signed by the parent of the participant.

All interviews were conducted at the hospital by a psychologist from the Department of Pediatric Hematology and Oncology. They were tape-recorded with the consent of the participants and transcribed. The mean duration of interviews was 46 min, with a minimum of 16 min and a maximum of 84 min.

### Interview Guide

The semi-structured interviews were designed as per the Schedule of Evaluation of Individual Quality of Life (Sei-QOL, O’Boyle et al., 1993), an individualized QoL assessment. Participants were asked to name the five most important dimensions of their QoL and to rate them by level of satisfaction and order of priority. In the context of our research, we did not use this instrument to specifically assess QoL but to encourage participants to think about their current everyday life in a global perspective, not only focusing on their illness and its current implications. Participants were invited to respond to the Sei-QOL at the beginning of the interview to help them talk about important domains of their current lives. Particular attention was paid to any reported current difficulty or challenge that the participants would relate to their past medical history. In such cases, the interviewer would use probes to invite the participant to reflect on past or current coping strategies used to overcome the difficulties.

### Data Analysis

Miles and Huberman (2003) suggested a two-step method of categorization, by predefining the main thematic categories and then allowing the emergence of subcategories from the transcripts. In alignment with our previously stated objectives, our initial thematic categories were defined as follows: (a) challenges and difficulties that the participants still experience due to their past illness and its complications and (b) resources or strategies that help them to overcome any difficulties. To ensure the validity of the findings and interpretations, the first and second author independently coded the first transcripts and met to discuss emerging themes. Then, the first author continued to code the transcripts and met the second author at regular intervals for further discussions. Moreover, in order to refine emerging categories and discuss implications for clinical practice, the psychologists from the Department of Pediatric Hematology and Oncology were invited to reflect and comment on the analysis process during a group discussion. The final categories and subcategories are presented in **Table 2**.

**Table 2:** Final categories and subcategories of the thematic analysis.

Categories	Subcategories
Challenges and difficulties that the	Physical consequences
participants still experience due to their	Psychosocial impact
past illness and its complications	Self-image
	Social withdrawal
	Sense of lack of choice
	Need for specific attention

Resources or strategies that help them	Talking about past illness
to overcome any difficulties.	Giving sense to past medical experiences
	Developing meaningful social relationships

## Results

### Current Challenges and Difficulties that the Participants Experience due to Their Past Illness and Its Complications

#### Physical Consequences

“It’s not the status of former patient that is problematic, it’s more the side effects that cause problems” (woman, 27.5 years old).

Physical consequences were among the main challenges reported by the participants as still impacting their QoL years after their illness and its treatment. No distinction was made between consequences due to the illness or due to the treatment. Participants reported several diseases (such as hepatitis C, hypertension, diabetes, thrombosis, etc.); the amputation of a member; short stature but also cognitive (fatigue, concentration, memory), motor (difficulties to walk), dermatological (vitiligo, scars), or ocular (cataract) consequences. Participants mentioned several problems at the same time or at different moments in their life, with the feeling that “there is always something wrong” (male adolescent, 16 years old). They underlined that they did not expect complications: “I thought that after my graft, it would be better, now I realize that I have lots of things that I did not think I would have” (male adolescent, 17.5 years old). Another principally reported consequence was sterility. The lack of information about the risk of sterility before and during their treatments emerged as an important issue.

#### Psychosocial Impact

##### Self-image

“In a way, I’m glad to be cured of the leukemia, but it’s not easy to live this life, with the visible scars I carry” (man, 22 years old).

Self-image appeared to have been affected by certain visible physical consequences. Short stature in particular was reported as impacting self-image. Participants underlined that this also compromised the development of affective and intimate relationships. Visible physical consequences were associated with a feeling of being judged by others which in turn affected self-image. Indeed, participants reported that people often expressed curiosity and astonishment by either questioning the former patient, looking away or making remarks, which emphasized for the participants the importance of appearance. The physical sequelae not only impacted participants but also the close family members and friends of these: “I went to an exposition, a books’ exposition with my boyfriend, I looked at a book and he told me “come, let’s leave,” I said “what happened?” And he told me “but did you not see the way she is looking at you?” People look at me stupidly and sometimes badly. Personally, I do not see them anymore because I’m used to seeing them looking at me but it bothers him [my boyfriend]” (woman, 27.5 years old).

##### Social withdrawal

Participants reported limitations in certain activities, such as physical exercise, driving, being around friends and having a romantic relationship. Moreover, finding a job that could be adapted to their abilities and limitations (such as fatigue, epilepsy, etc.) can emerge as an important challenge.

In addition, concern from others seemed to discourage participants from actively participating in social activities. For example, participants reported that others (mostly family members or friends) were anxious about the physical risks of certain activities, such as going to a bar, going on holiday, or trying a new activity. Sometimes close family members or friends recommended against or even prohibited participants from doing these activities. Other times, the former patient prevented himself from doing these activities in order not to make friends or family anxious: “I feel like dancing or having a drink but I refrain from doing it because doing activities with me makes them [my friends] a little bit anxious that something may happen to me (such as bleedings). They invite me to go with them, I do it sometimes but rarely” (man, 21 years old).

##### Sense of lack of choice

Feeling restricted in their options with regards to studies, work, leisure activities or life projects appeared as a key issue. Choices with regards to studies were felt to have been affected by long-term consequences, such as cognitive or physical difficulties. The fear of relapse or complications or simply bad memories prevented pursuing scholarly activities: “Well, maybe I would have liked to study more before becoming a primary school teacher. But, I never gave myself this opportunity actually. I closed some doors deliberately because I said to myself: and what if I fall ill again?” (woman, 30 years old).

Fear also influenced family members, who prevented patients from realizing their projects: “I regret a little bit not having the liberty to make my choices. Even if I studied hairdressing and I was in pain, never mind. I would have stopped. But I would have liked to let myself do what I wanted” (woman, 32.5 years old).

Nevertheless, despite this lack of choice, participants expressed contentment with their second choice and no longer harbored any regrets.

##### Need for specific attention

“I’m like everyone else, I don’t know, I’m a human being. I speak like everyone else, I breathe like everyone else, I listen like everyone else, I’m not an alien” (man, 20 years old).

Participants had some special needs, such as the need to receive attention: “When we are ill, we are surrounded, pampered. […] And now I need others to be concerned about me and that’s why I am telling you that I find myself really selfish” (woman, 30 years old).

The need to be considered as a normal person and not to feel a difference with other people also emerged as an important issue: “Sometimes, when we say that we had a leukemia, the others look at you as if you are an insect or a parasite that is going to come to upset the life of other people” (woman, 19 years old).

Moreover, participants reported that when people knew that they had a cancer, they often changed their attitudes, by over-protecting them, taking pity on them, being afraid, etc.: “My status is a status where either people protect me too much […] or it is as if I did not exist. It’s really boring and I would like people to tell me: well you are equal, period!” (woman, 27.5 years old).

These attitudes have often led AYA survivors to avoid speaking about their past illness. When they overcome the illness, they want to be considered for what they are today, for their current competences and not for their past experiences.

### Resources or Strategies that Help AYA Survivors to Overcome Their Difficulties

The psychosocial and physical impacts of their past illness, its treatment and its side-effects led AYA survivors to adapt their environment and to develop resources to help them overcome their difficulties. In this section, we report the principle resources or strategies reported by participants to live as well as possible with their past medical experiences, namely (a) talking about past illness, (b) giving sense to past illness, and (c) developing meaningful social relationships.

#### Talking about Past Illness

Discussing past illness seems to depend on the interlocutor. At work, if they were not obliged to talk about their illness because of current complications, many AYA survivors did not talk about their illness to their employer and colleagues. This was mainly due to fear of being treated differently or of not being hired: “It’s not what I would like to share with other people because I want to be like everyone else. I don’t want to be employed because of that but for my competences” (woman, 23 years old).

Discussing their past illness with close family and friends was sometimes considered as a need and other times as a taboo subject for the participant and/or for the family: “With mummy, there is an age from which I stopped talking with her because I feel she’s in pain. She would like, well she would like… She always says <<forget, stop talking about that>>” because it hurts her. But there were some moments where I needed to speak with her about it. Now, because I restrained myself from talking about it for a few years, I really want to shout it all over the world” (woman, 32 years old).

Speaking about past illness is rather executed on demand than spontaneously. The reasons to talk about their illness included:

(a) The need to understand: “I just talked about it with my parents a short time ago. I learned a lot of things that I did not remember. I’m ready to speak about that sometimes to know how it happened and try to understand” (man, 20 years old);
(b) the desire to respond to questions: “I prefer to be asked and I will respond without problem, honestly but otherwise I would not talk about that openly like that, just for my pleasure” (man, 21 years old);
(c) the desire to help other people: “In my entourage, a lot of persons are ill. Sometimes, I describe to them what I experienced. And maybe it can reassure them and at the same time, it releases me a little bit, I don’t know” (woman, 21 years old);
(d) the wish to explain visible physical remnants (height, scars, epilepsy, medication, etc.): “I said, for example, that I had had leukemia and that after that I became epileptic. And then sometimes, when someone tells me that I’m short, I tell them that it’s because of the illness when I was younger” (man, 22 years old).

The reasons for not talking about illness were the desire not to make others worried or the desire to move on to something else or the desire not to be seen as complaining.

We also noted that there were changes over time in the way people discuss their medical experiences, speaking about it more now than before or vice versa.

#### Giving Sense to Past Medical Experiences

“It’s a step in life, it’s an experience” (male adolescent, 16 years old).

One way that participants cope with the past illness is to give it a sense, such as describing how their illness made them mature quicker than their peers: “The illness has made me grow up. The illness I had, I know very well that it’s a part of my life that made me grow up a lot. I learned a lot of things” (man, 20 years old).

Considering the past illness as an enriching experience for them but also for their close family emerged as an important topic of discussion: “Enriched, yes because it brings another way to see things. But I think that it was not only enriching for me. It enriches all the people who are around me too. In a way, we never overcome that alone… I think of that as an experience that can be enriching for everybody. With the assumption that we are cured of course because we cannot say that people who die find it interesting” (man, 29 years old).

Giving a sense to their past illness was a way to integrate it in their current life and to recognize that in spite of all the difficulties they faced, it allowed them to become who they are: “I tell myself that it’s a part of me. In my opinion I would not be who I am if I had not had that. I’m sure. So I want to say it’s a part of me and I always live with it. And I think it has influenced some of my choices, but that’s it” (man, 28.5 years old).

However, questioning the reasons for such an experience and wondering what former patients would have become without the illness seemed to hinder the path to acceptance: “Being born with a problem, it is not easy to accept. And that is true for many people. Having something later in life, even if it’s not something stupid I did like a car crash or something else that I would blame myself personally for. Here, I cannot blame anybody. I’m in it, I mean that I know what I was and I see what I became and that’s it! And the question that we ask ourselves is <<what would I have become if I had not had that?>>” (man, 21 years old).

#### Developing Meaningful Social Relationships

Being surrounded by their family and friends emerged as important issue. Social support was important during the illness and its treatments but it continued to be a resource for the former patients in their current life: “It’s important to have friends. When you have a moment of depression or anything else, they come to lift your spirits. Parents also come to lift your spirits; all these tricks make us feel better” (man, 20 years old).

Moreover, the need to develop meaningful social relationships was partly explained by their past experience and the desire to be better surrounded if something difficult had to happen. It is a way to anticipate eventual future problems, based on past experiences: “Maybe the need to have lots of friends everywhere is because, if something had to happen to me tomorrow, even if I know one hundred persons, there would be maybe 3 who would stay tomorrow evening” (man, 20.5 years old).

## Discussion

The aim of the present study was to investigate qualitatively what difficulties adult survivors of childhood cancer and treatment with HSCT still face in their life due to their past illness and what strategies they adopted to cope with their past cancer, treatment with a HSCT and its long-term effects. In order to explore these themes, we conducted a descriptive and exploratory study based on individual in-depth interviews.

Initial findings indicated that participants mainly mentioned physical and psychosocial difficulties. Moreover, the results showed that when participants evoked these physical or psychosocial consequences, they did not differentiate between the causes of these. Nevertheless, these complications can be attributed to several aspects of the management of a childhood cancer from the initial diagnosis for which the HSCT is performed, the conditioning regimen, and the treatment of the complications of the HSCT, such as GvHD (Baker et al., 2010a). The types of physical consequences reported by former participants concurred with those described in the literature, such as endocrine, neurological, or neuropsychological problems (Steffens et al., 2008; Baker et al., 2010a; Benmiloud et al., 2010), as well as infertility (Hammond et al., 2007; Jadoul et al., 2011). Most of the participants reported that they were not informed of the risk of sterility. The reasons for this lack of awareness were rarely evoked and could be multiple. Indeed, it could be that (a) care providers did not give enough information because the child was too young or (b) parents did not want to inform their child to avoid deception or (c) patients themselves were informed but preferred masking this information.

Concerning the psychosocial impact of pediatric cancer and its treatments, previous studies show that even if adult survivors of HSCT seem to maintain a satisfactory QoL, many report a number of psychological and social problems (Löf et al., 2009; Sanders et al., 2010). However, these long-term sequelae are often categorized in general terms and do not provide any granularity to understanding the difficulties that AYA survivors meet in their daily life. Eiser et al. (2000) reported, in a systematic review, that there were few differences in the psychological consequences of those surviving pediatric cancer when authors used standardized measures. Differences appeared when authors used interviews in order to comprehend the psychological impact of childhood cancer, highlighting for example an affected body image, more limitation of activities, and less risky behaviors in alcohol use in survivors. These results are in line with the review of Patenaude and Kupst (2005). They showed that social difficulties (for example, employment challenges or poor relationships) and impaired self-esteem or self-concept were two important long-term consequences of pediatric cancer. These results largely agree with the categories that emerged from our interviews, especially with relation to self-image and social withdrawal.

Our results highlight a need for specific attention among the study population, which along with the perception of lack of choices, has rarely been addressed in the literature. The need to be considered as a normal person and the feeling that choices were limited had implications on social life, self-image, and distress levels. We consider that addressing those aspects of adjustment would be worthwhile to promote the well-being of AYA survivors.

Our study also explored adaptation strategies and the resources used by AYAs to live with their past illness and its consequences. Three main resources emerged from the interviews: (a) talking about past illness, (b) giving sense to past illness, and (c) developing meaningful social relationships. Such strategies can fulfill different functions and are not in essence adapted or non-adapted. Depending on the context (the moment, the duration, the social environment, etc.), some may be functional or not. It appears that these strategies can have a social and an individual function.

To illustrate this further, among individual functions, talking about illness could help the former patient to understand the illness and its consequences, while not talking about illness helped other participants to move on to something else. There was also a social impact of this decision to talk or not to talk, such as responding to questions, helping other people, protecting others from experiencing worry, etc. Such examples demonstrate that flexibility and adaptability are essential to successful coping rather than the exclusive use of one strategy (Bonanno et al., 2004).

The concept of flexibility is not only applicable to the expression of emotions rather it is a more general perspective to consider the adaptation of people and to overcome a categorical perspective, such as the classic dual coping strategies categories (e.g., problem- vs. emotion-focused coping, Lazarus and Folkman, 1984 or approach vs. avoidance coping, Roth and Cohen, 1986).

Adolescent and young adult survivors used coping strategies such as talking or not talking about past illness, giving sense to past illness, and seeking social support (especially with parents and close friends, as showed by Trask et al., 2003). However, some of them were less able to adjust their behaviors to the contextual demands and opportunities and to the (non) efficacy of their initial strategies, which could be detrimental for psychosocial well-being (Bonanno and Burton, 2013).

### Limitations and Perspectives

Firstly, although this study allowed participants to evoke their past illness and its complications with a professional, the risk of this type of interview is that it leads to the <<focusing illusion>> (Schkade and Kahneman, 1998). Indeed, we encouraged participants to talk about their life and their QoL in general but being in the hospital, in front of a pediatric psychologist, their attention was probably focused only on a part of their life devoted to their past illness and its complications. It is likely that in another context participants would make fewer links between their current life and their past medical experiences.

Secondly, the context of the research could have an influence on the participation rate and on the duration of the interviews. The interview with the psychologist was integrated as an option in a larger multidisciplinary research, which prevents some voluntary participants from taking part in the psychological interview because they did not have the time to combine the different appointments. Moreover, because of the intensity of medical program (number of appointments, duration of some medical examinations, etc.), some of the interviews had to be shortened to respect the medical program, which could partly explain the difference in the duration of the interviews (range between 16 and 84 min).

Thirdly, since there was a significant time period between the diagnosis of cancer and the interviews, we should bear in mind the changes in treatments; the development of new interventions, such as preservation of fertility, but also changes to communication between the medical team and children and their families. From a medical perspective, different authors showed over the last 25 years an increase in HSCT complexity but also major advances in supportive care and a refinement of donor selection (Mateos et al., 2013; Brissot et al., 2016; Svenberg et al., 2016). All these developments have probably impacted on the physical and psychosocial consequences following HSCT after a pediatric cancer. Therefore, it is likely that children treated today will not encounter the same physical and psychosocial consequences later in life as those reported here.

Moreover, the time period between the diagnosis and the interviews could be really different between participants, which could have an influence on their experiences. Receiving an allogeneic or an autologous transplantation could also have different psychosocial consequences on patients and their families. To illustrate, Packman et al. (2010) highlighted, in their review, that the distress of parents could be greater when one of their healthy children is the donor. The donating experience could also differently affect donor and non-donor siblings. Donor siblings seem to show more internalizing (anxiety and affected self-esteem) and externalizing problems (behavioral difficulties), but also more adaptive skills in school than non-donor siblings (Packman et al., 2010). However, from a qualitative perspective, we aimed at describing experiences of AYA survivors of HSCT. We did not seek to conduct comparisons between genders, ages, types of HSCT (allogeneic vs. autologous), etc. These questions would be worthy of further consideration in order to take into account moderators such as contextual and individual characteristics that could have an impact on physical or psychosocial adjustment.

Finally, this research was based on AYA survivors’ experiences. However, from a longitudinal perspective, it could be worthwhile to explore parental perceptions regarding not only the development of their child, but also their functioning as parents. Indeed, some studies showed that coping strategies and adjustment of children were related to those of their parents and that parents can promote certain coping strategies in their child with cancer (e.g., Sanger et al., 1991; Kupst et al., 1995; Hildenbrand et al., 2014). Further studies could investigate whether these links remain present even when former patients become adults and independent.

### Implications for Clinical Practice

Our findings have implications for each practitioner involved in the treatment and the support of children with a cancer or a past experience of cancer and their family. The importance of informing youths, according to their needs, as well as capacities, regarding potentially multifaceted physical consequences of their illness and its treatment (number, frequency, intensity of the side-effects) has been highlighted. The aim is to prepare them for the future and to support their consideration of all the treatment options, such as fertility preservation, etc. The communication between care providers and patients in oncology is becoming a major field of interest and research (Stiefel et al., 2009). However, most studies are focused on the communication skills of professionals and only a few studies are patient-oriented (Barth and Lannen, 2011). Moreover, to our knowledge, none targets pediatric patients and their families. Therefore, it seems really worthwhile to develop research projects based on the communication between providers of care and pediatric patients and their families.

Finally, better understanding how AYA survivors live with their past illness has worthwhile implications for developing individualized interventions during treatment and improving long-term psychosocial interventions. As recommended by Children’s Oncology Group (2008), the present study also suggests developing adapted psychosocial follow-ups in the years after the remission of cancer in order to facilitate early identification of resources and vulnerabilities of AYA survivors.

## Author Contributions

All authors listed, have made substantial, direct and intellectual contribution to the work, and approved it for publication.

## Conflict of Interest Statement

The authors declare that the research was conducted in the absence of any commercial or financial relationships that could be construed as a potential conflict of interest.

The reviewer SB and handling Editor declared their shared affiliation, and the handling Editor states that the process nevertheless met the standards of a fair and objective review.

## References

[B1] Armenian S. H. Sun C.-L. Kawashima T. Arora M. Leisenring W. Sklar C. A. (2011). Long-term health-related outcomes in survivors of childhood cancer treated with HSCT versus conventional therapy: a report from the Bone Marrow Transplant Survivor Study (BMTSS) and Childhood Cancer Survivor Study (CCSS). *Blood* 118 1413–1420. 10.1182/blood-2011-01-331835 21652685 PMC3152502

[B2] Baker K. S. Armenian S. Bhatia S. (2010a). Long-term consequences of hematopoietic stem cell transplantation: current state of the science. *Biol. Blood Marrow Transplant.* 16 S90–S96. 10.1016/j.bbmt.2009.09.017 19782145 PMC2832710

[B3] Baker K. S. Bresters D. Sande J. E. (2010b). The burden of cure: long-term side effects following hematopoietic stem cell transplantation (HSCT) in children. *Pediatr. Clin. North Am.* 57 323–342. 10.1016/j.pcl.2009.11.008 20307723

[B4] Barth J. Lannen P. (2011). Efficacy of communication skills training courses in oncology: a systematic review and meta-analysis. *Ann. Oncol.* 22 1030–1040. 10.1093/annonc/mdq441 20974653

[B5] Beauloye V. Steffens M. Zech F. Vermylen C. Maiter D. (2013). Characterization of insulin resistance in young adult survivors of childhood acute lymphoblastic leukaemia and non-Hodgkin lymphoma. *Clin. Endocrinol.* 78 790–798. 10.1111/cen.12047 22967316

[B6] Benmiloud S. Steffens M. Beauloye V. De Wandeleer A. Devogelaer J.-P. Brichard B. (2010). Long-term effects on bone mineral density of different therapeutic schemes for acute lymphoblastic leukemia or non-Hodgkin lymphoma during childhood. *Horm. Res. Paediatr.* 74 241–250. 10.1159/000313397 20395671

[B7] Bonanno G. A. Burton C. L. (2013). Regulatory flexibility an individual differences perspective on coping and emotion regulation. *Perspect. Psychol. Sci.* 8 591–612. 10.1177/1745691613504116 26173226

[B8] Bonanno G. A. Papa A. Lalande K. Westphal M. Coifman K. (2004). The importance of being flexible the ability to both enhance and suppress emotional expression predicts long-term adjustment. *Psychol. Sci.* 15 482–487. 10.1111/j.0956-7976.2004.00705.x 15200633

[B9] Brissot E. Rialland F. Cahu X. Strullu M. Corradini N. Thomas C. (2016). Improvement of overall survival after allogeneic hematopoietic stem cell transplantation for children and adolescents: a three-decade experience of a single institution. *Bone Marrow Transplant.* 51 267–272. 10.1038/bmt.2015.250 26642337

[B10] Cairo M. Heslop H. (2008). Pediatric blood and marrow transplantation: state of the science. *Bone Marrow Transplant.* 41 97 10.1038/sj.bmt.1705992 18231112

[B11] Children’s Oncology Group (2008). *Long-term Follow-up Guidelines for Survivors of Childhood, Adolescent, and Young Adult Cancers.* Arcadia, CA: Children’s Oncology Group.

[B12] Clarke S. Eiser C. Skinner R. (2008). Health-related quality of life in survivors of BMT for paediatric malignancy: a systematic review of the literature. *Bone Marrow Transplant.* 42 73–82. 10.1038/bmt.2008.156 18500369

[B13] Copelan E. A. (2006). Hematopoietic stem-cell transplantation. *N. Engl. J. Med.* 354 1813–1826. 10.1056/NEJMra052638 16641398

[B14] Eiser C. Hill J. J. Vance Y. H. (2000). Examining the psychological consequences of surviving childhood cancer: systemic review as a research method in pediatric psychology. *J. Pediatr. Psychol.* 25 449–460. 10.1093/jpepsy/25.6.449 10980049

[B15] Gatta G. Botta L. Rossi S. Aareleid T. Bielska-Lasota M. Clavel J. (2014). Childhood cancer survival in Europe 1999–2007: results of EUROCARE-5—a population-based study. *Lancet Oncol.* 15 35–47. 10.1016/S1470-2045(13)70548-5 24314616

[B16] Hammond C. Abrams J. R. Syrjala K. L. (2007). Fertility and risk factors for elevated infertility concern in 10-year hematopoietic cell transplant survivors and case-matched controls. *J. Clin. Oncol.* 25 3511–3517. 10.1200/JCO.2007.10.8993 17646668 PMC6391980

[B17] Hildenbrand A. K. Alderfer M. A. Deatrick J. A. Marsac M. L. (2014). A mixed methods assessment of coping with pediatric cancer. *J. Psychosoc. Oncol.* 32 37–58. 10.1080/07347332.2013.855960 24428250 PMC3929263

[B18] Jadoul P. Anckaert E. Dewandeleer A. Steffens M. Dolmans M.-M. Vermylen C. (2011). Clinical and biologic evaluation of ovarian function in women treated by bone marrow transplantation for various indications during childhood or adolescence. *Fertil. Steril.* 96 126–133.e3 10.1016/j.fertnstert.2011.03.108 21550046

[B19] Kupst M. J. Bingen K. (2006). “Stress and coping in the pediatric cancer experience,” in *Comprehensive Handbook of Childhood Cancer and Sickle Cell Disease: A Biopsychosocial Approach* ed. Brown R. T. (New York, NY: Oxford University Press) 35–52.

[B20] Kupst M. J. Natta M. B. Richardson C. C. Schulman J. L. Lavigne J. V. Das L. (1995). Family coping with pediatric leukemia: ten years after treatment. *J. Pediatr. Psychol.* 20 601–617. 10.1093/jpepsy/20.5.601 7500233

[B21] Lazarus R. S. Folkman S. (1984). *Stress, Appraisal, and Coping.* Berlin: Springer publishing company.

[B22] Löf C. Winiarski J. Giesecke A. Ljungman P. Forinder U. (2009). Health-related quality of life in adult survivors after paediatric allo-SCT. *Bone Marrow Transplant.* 43 461–468. 10.1038/bmt.2008.338 18936733

[B23] Mateos M. K. O’brien T. A. Oswald C. Gabriel M. Ziegler D. S. Cohn R. J. (2013). Transplant-related mortality following allogeneic hematopoeitic stem cell transplantation for pediatric acute lymphoblastic leukemia: 25-year retrospective review. *Pediatric Blood Cancer* 60 1520–1527. 10.1002/pbc.24559 23733511 PMC3798104

[B24] Miles M. B. Huberman A. M. (2003). *Analyse des Données Qualitatives.* Paris: De Boeck Supérieur.

[B25] O’Boyle C. McGee H. Hickey A. Joyce C. Browne J. O’Malley K. (1993). *The Schedule for the Evaluation of Individual Quality of Life (SEIQoL)*. *Administration Manual.* Available at: http://epubs.rcsi.ie/psycholrep/39/

[B26] Packman W. Weber S. Wallace J. Bugescu N. (2010). Psychological effects of hematopoietic SCT on pediatric patients, siblings and parents: a review. *Bone Marrow Transplant.* 45 1134–1146. 10.1038/bmt.2010.74 20383219

[B27] Parsons S. K. Phipps S. Sung L. Baker K. S. Pulsipher M. A. Ness K. K. (2012). NCI, NHLBI/PBMTC First International Conference on Late Effects after Pediatric Hematopoietic Cell Transplantation: health-related quality of life, functional, and neurocognitive outcomes. *Biol. Blood Marrow Transplant.* 18 162–171. 10.1016/j.bbmt.2011.12.501 22155139 PMC3258340

[B28] Patenaude A. F. Kupst M. J. (2005). Psychosocial functioning in pediatric cancer. *J. Pediatr. Psychol.* 30 9–27. 10.1093/jpepsy/jsi012 15610981

[B29] Phipps S. Dunavant M. Lensing S. Rai S. N. (2005). Psychosocial predictors of distress in parents of children undergoing stem cell or bone marrow transplantation. *J. Pediatr. Psychol.* 30 139–153. 10.1093/jpepsy/jsi002 15681309

[B30] Roth S. Cohen L. J. (1986). Approach, avoidance, and coping with stress. *Am. Psychol.* 41 813–819. 10.1037/0003-066X.41.7.813 3740641

[B31] Sanders J. E. Hoffmeister P. A. Storer B. E. Appelbaum F. R. Storb R. F. Syrjala K. L. (2010). The quality of life of adult survivors of childhood hematopoietic cell transplant. *Bone Marrow Transplant.* 45 746–754. 10.1038/bmt.2009.224 19718073 PMC2850957

[B32] Sanger M. S. Copeland D. R. Davidson E. R. (1991). Psychosocial adjustment among pediatric cancer patients: a multidimensional assessment. *J. Pediatr. Psychol.* 16 463–474. 10.1093/jpepsy/16.4.463 1941426

[B33] Schkade D. A. Kahneman D. (1998). Does living in California make people happy? A focusing illusion in judgments of life satisfaction. *Psychol. Sci.* 9 340–346. 10.1111/1467-9280.00066

[B34] Siegel R. DeSantis C. Virgo K. Stein K. Mariotto A. Smith T. (2012). Cancer treatment and survivorship statistics, 2012. *CA Cancer J. Clin.* 62 220–241. 10.3322/caac.21149 22700443

[B35] Steffens M. Beauloye V. Brichard B. Robert A. Alexopoulou O. Vermylen C. (2008). Endocrine and metabolic disorders in young adult survivors of childhood acute lymphoblastic leukaemia (ALL) or non-Hodgkin lymphoma (NHL). *Clin. Endocrinol.* 69 819–827. 10.1111/j.1365-2265.2008.03283.x 18429947

[B36] Stiefel F. Barth J. Bensing J. Fallowfield L. Jost L. Razavi D. (2009). Communication skills training in oncology: a position paper based on a consensus meeting among European experts in 2009. *Ann. Oncol.* 21 204–207. 10.1093/annonc/mdp564 20026475

[B37] Svenberg P. Remberger M. Uzunel M. Mattsson J. Gustafsson B. Fjaertoft G. (2016). Improved overall survival for pediatric patients undergoing allogeneic hematopoietic stem cell transplantation–A comparison of the last two decades. *Pediatr. Transplant.* 20 667–674. 10.1111/petr.12723 27251184

[B38] Trask P. C. Paterson A. G. Trask C. L. Bares C. B. Birt J. Maan C. (2003). Parent and adolescent adjustment to pediatric cancer: associations with coping, social support, and family function. *J. Pediatr. Oncol. Nurs.* 20 36–47. 10.1053/jpon.2003.5 12569433

[B39] Zecca M. Prete A. Rondelli R. Lanino E. Balduzzi A. Messina C. (2002). Chronic graft-versus-host disease in children: incidence, risk factors, and impact on outcome. *Blood* 100 1192–1200. 10.1182/blood-2001-11-0059 12149197

